# Pre-gestational stress alters stress-response of pubertal offspring rat in sexually dimorphic and hemispherically asymmetric manner

**DOI:** 10.1186/1471-2202-14-67

**Published:** 2013-07-08

**Authors:** Yuejun Huang, Sihong Chen, Hongwu Xu, Xiaochan Yu, Huihong Lai, Guyu Ho, Qingjun Huang, Xuechuan Shi

**Affiliations:** 1 Transforming Medical Center, Second Affiliated Hospital of Medical College of Shantou University, North Dongxia Rd, Shantou, Guangdong 515041, China; 2 Department of Pediatrics, Second Affiliated Hospital of Medical College of Shantou University, North Dongxia Rd, Shantou, Guangdong 515041, China; 3 Department of Neurosurgery, Second Affiliated Hospital of Medical College of Shantou University, North Dongxia Rd, Shantou, Guangdong 515041, China; 4 Joint Lab of Biological Psychiatry, Mental Health Center of Shantou University, Taishan Rd, Shantou, Guangdong 515041, China

**Keywords:** Stress, Dopamine, Medial prefrontal cortex, Dopamine transporter, Norepinephrine transporter, Catechol-O-methyltransferase

## Abstract

**Background:**

There is increasing evidence that maternal stress may have long-term effects on brain development in the offspring. In this study, we examined whether pre-gestational stress might affect offspring rats on the medial prefrontal cortical (mPFC) dopaminergic activity in response to acute stress in puberty and if so, whether such effects exhibited hemispheric asymmetry or sexual dimorphism.

**Results:**

We used behavioral tests to assess the model of chronic unpredictable stress (CUS). We found that the activity in the open field test and sucrose intake test were lower for maternal rats in the CUS group than those in the control group. Offspring rats in the CUS group floated more and swam or climbed less as compared to the offsprings in the control group in the forced swimming test. The floating time was longer and swimming or climbing time was shorter in the female offspring rats than those in the males. Serum corticosterone and corticotrophin-releasing hormone levels were significantly higher for CUS maternal rats and their offsprings than the respective controls. The ratio of dihydroxy-phenyl acetic acid (DOPAC) to dopamine (DA), DA transporter (DAT), norepinephrine transporter (NET) were lower in the mPFC of offspring rats in the CUS group than the control group. Levels of catechol-O-methyltransferase (COMT) in the left mPFC of female offspring rats and in the right mPFC of both female and male offspring rats were lower in the CUS group than those in the controls, but there was no difference in the left mPFC of male offspring between the CUS and control groups. DOPAC, the ratio of DOPAC to DA, NET and COMT were lower in the right mPFC than in the left mPFC of offspring rats in the CUS group. The ratio of DOPAC to DA in the right mPFC was lower in the female offspring rats than male offspring rats in the CUS group. The NET and COMT levels in both left and right mPFC were lower in the female offspring rats than those of the male offsprings in the CUS group.

**Conclusion:**

Our data provide evidence that the effect of pre-gestational stress on the mPFC dopaminergic activity in response to acute stress exhibited hemispheric asymmetry and sexual dimorphism in the pubertal offspring rats.

## Background

There is increasing evidence that maternal stress may have long-term effects on brain development in the offspring [1] and, consequently, increase the risk for psychiatric disorders, such as depression, schizophrenia, attention-deficit hyperactivity disorder and autism [2,3].

The most extensively studied system susceptible to stress is the hypothalamus–pituitary–adrenal (HPA) axis. This neuroendocrine axis regulates a variety of metabolic processes by secreting glucocorticoids (GCs) in response to stress [4,5]. Elevated GCs exert negative feedback on the hypothalamus either directly or via other brain regions [5,6]. In the rat, exposure to a high level of GCs during pregnancy sensitises the HPA activity to stress later life [7]. The medial prefrontal cortex (mPFC) is involved in regulating the behavioral, neuroendocrine, and autonomic responses to stress [8-10] and also plays a pivotal role in so-called executive functions. The development of the prefrontal cortex is considered to be the last area of the cortex to mature, which continues to develop until early adulthood [11]. This delay has been suggested to be associated with behaviours such as impulsivity in adolescence [12,13]. Adolescence is a critical stage for the development of emotional maturity and diverse forms of psychopathology [11]. Changes of dopaminergic activity in the mPFC of pubertal rats could be due to various stresses during development, where the dopaminergic system plays a modulatory role by optimising the activity of mPFC neurons [14,15]. Moreover, the activity of the mPFC dopaminergic system is positively regulated by GCs [16,17]. Not surprisingly, disorders of higher executive processes often reflect disruptions in mPFC dopamine (DA)-mediated function. Stressors potently stimulate DA release in the mPFC [18]. Perhaps less well known is that the DA inputs to the left and right mPFC are asymmetric to stress responses [19,20]. Furthermore, the development of such hemispheric asymmetries in mPFC can be altered by perinatal factors [21]. These findings, along with the evidence derived from clinical observations, have led to the suggestion that abnormal lateralisation of mPFC DA-mediated function may increase the vulnerability to a range of stress-related psychopathologies [22].

Although some studies [4-6] have examined the effects of pre-gestational stress on gene or protein expression in the hippocampus of both female and male offspring, little research has devoted to the relationship between the sex differences in behaviors, HPA axis activity and effects of pre-gestational stress on the dopaminergic system in the mPFC of pubertal offspring rats. To address these questions, we sought to determine whether pre-gestational stress might contribute to individual differences in the mPFC dopaminergic activity in response to stress during puberty and if so, whether such differences possessed hemispheric asymmetry or sexual dimorphism. We used a short-term acute stress procedure, such as the forced swimming test (FST) to examine the effect of pre-gestational stress on behavioral responses to acute stress and subsequent changes of the dopaminergic activity in the mPFC of offspring rats at postnatal day (PND) 30. Changes in the HPA-axis activity of maternal rats and their offsprings in response to stress were assessed by serum corticosterone (COR) and corticotrophin-releasing hormone (CRH). In addition, we measured the dopaminergic activity by assessing DA, its metabolite dihydroxy-phenyl acetic acid (DOPAC) and the ratio of DOPAC to DA. To gain the mechanism understanding of any changes in the dopaminergic system, levels of DA transporter (DAT), norepinephrine transporter (NET) and catechol-O-methyltransferase (COMT) were evaluated.

Additionally, the impact of pre-gestational CUS on the behavioral changes inducing by FST was examined in the offspring rats of both sexes. FST, apart from a useful screening test for antidepressants, has also been considered a stressful condition to induce behavioral changes, which are indices of “depressive-like” symptomatology [23]. Therefore, we hypothesized that the application of FST on pubertal male and female offspring rats born to pre-gestational CUS mothers would yield more information about sex differences in response to the acute stressor (i.e.,FST).

## Methods

### Animals

Adult male (n = 10) and female (n = 20) Sprague–Dawley rats (approximately 8 weeks old; 250 g-300 g and 200 g-250 g, respectively) were provided by the Animal Centre of Shantou University. Female rats were nulliparous. Before the beginning of any stress procedures, female rats were divided into 2 groups for treatment: control (n = 8) and chronic unpredictable stress (CUS) (n = 12). Female rats were housed singly, and male rats were housed in pairs in plastic, non-transparent cages (60 cm × 40 cm × 25 cm) in separate rooms under controlled 12-h light/12-h dark conditions (lights on at 08:00) and temperature (24°C). All rats had free access to food and water throughout the experiments, unless specified by the experimental procedure. Female rats exhibited a normal oestrous cycle (4–5 days of oestrous) before the start of the experiment, with equal distribution of different stages of the oestrous cycle. The oestrous cycle was reconfirmed during the last week of CUS with the use of vaginal smears.

All animal experiments were reviewed and approved by the Medical Animals Care and Welfare Committee of Shantou University Medical College (Shantou, China). All studies were carried out in accordance with the US National Institutes of Health Guide for the Care and Use of Laboratory Animals (NIH Publications No. 80–23 revised 1996). Every effort was made to minimise the number of animals used and reduce suffering.

### CUS procedure

Body weight of maternal rats was measured before the start of CUS (W0) and once a week (W1, W2, W3) during CUS for 3 weeks. The CUS procedure, sucrose intake test and open field test were as we previously described [4,5]. The schedule is shown in Table 1[5] and Table 2[5].

**Table 1:** The adaptation period of sucrose intake test

**Time**	**Course of the experiment**
The 1st day 8:00	Food and water deprivation for 24 h
The 2nd day 8:00	One-bottled-sucrose test sessions (last 1 h)
An interval of 3 days	
The 6th day 8:00	Food and water deprivation for 24 h
The 7th day 8:00	One-bottled-sucrose test sessions (last 1 h)
An interval of 3 days	
The 11th day 8:00	Food and water deprivation for 24 h
The 12th day 8:00	One-bottled-sucrose test sessions (last 1 h)
An interval of 3 days	
The 16th day 6:30	Weighed body weight and open field test
The 16th day 8:00	Food and water deprivation for 24 h
The 17th day 8:00	One-bottled-sucrose test sessions (last 1 h)
The 17th day 9:00	The beginning of CUS procedure

**Table 2:** The CUS procedure

**Time**	**Course of the experiment**
The 1st day 9:00	Cage rocking (5 times per second) for 15 min
The 2nd day 9:00	Swimming in cold (4°C) water for 5 min
The 3rd day 9:00	Soiled cage (250 ml of tap water into the sawdust bedding) for 24 h
The 4th day 9:00	Lights on overnight
The 5th day 9:00	Cage tilting for 24 h
The 6th day 9:00	Restraint for 12 h
The 7th day 8:00	Food and water deprivation for 24 h
The 8th day 8:00	One-bottled-sucrose test sessions (last 1 h)
The 8th day 9:00	Soiled cage for 24 h
The 9th day 9:00	Electric stimulus (1.0 mA, each time for 1 second, 10 times per minute) for 5 min
The 10th day 9:00	Elevated temperature (40°C) for 15 min
The 11th day 9:00	Cage rocking for 15 min
The 12th day 9:00	Lights on overnight
The 13th day 9:00	Restraint for 12 h
The 14th day 8:00	Food and water deprivation for 24 h
The 15th day 8:00	One-bottled-sucrose test sessions (last 1 h)
The 15th day 9:00	Lights on overnight
The 16th day 9:00	Swimming in cold water for 5 min
The 17th day 9:00	Restraint for 12 h
The 18th day 9:00	Electric stimulus for 5 min
The 19th day 9:00	Cage rocking for 15 min
The 20th day 9:00	Soiled cage for 24 h
The 21th day 9:00	Cage tilting for 24 h (The CUS procedure finish)
The 22th day 6:30	Weighed body weight and open field test
The 22th day 8:00	Food and water deprivation for 24 h
The 23th day 8:00	One-bottled-sucrose test sessions (last 1 h)

### The open field test

The method of the open field test was that described by us previously [4]. Briefly, all female rats were transferred to the test room one hour before testing for acclimatisation. The test room had controlled sound insulation, light insulation and temperature (24°C) conditions. The open field consisted of a square arena (60 cm × 60 cm), with a white floor divided into 36 squares (10 cm × 10 cm), enclosed by continuous 25-cm-high walls made of black plastic. In this test, the 20 squares adjacent to the wall represent a protected field, named the “peripheral arena”, while the other 16 squares represent an exposed field or the “central arena”. The test was initiated by placing a single rat in the middle of the arena and letting it move freely for 5 min. The movement of rats was continuously videotaped by a video camera placed over the structure and then recorded using a continuous sampling method. The videotapes were analysed by a video tracking system (Ethovision, Noldus Information Technology, The Netherlands). The arena was carefully cleaned with alcohol and rinsed with water after each test.

### Pregnancy of female rats

When the CUS procedure was finished (24 h after the last stressor), all female rats were housed in pairs with a male for 1 week for mating, before which blood samples were collected for CRH and COR detection. The day on which sperm was observed in vaginal smears was designated as embryonic day 0. All control rats (n = 8) and 10 of the 12 CUS rats became pregnant. Nest material was provided for gestational female rats, which were housed singly and undisturbed. The day of delivery was designated as PND 0. After 24 hours blood samples were collected again for post-delivery CRH and COR detection. All blood samples were taken from the vena cava caudalis between 10:00 and 12:00. There were 90 offspring rats (46 females and 44 males) from maternal rats of the control group and 96 offspring (47 females and 49 males) from the CUS group. In order to minimize the number of animal used and to reduce suffering, we randomly selected 6 offspring rats (3 females and 3 males) from each maternal rat for the following experiments, including FST, measurement of serum COR and CRH levels, HPLC and western blot analysis of brain tissue samples of offspring rats.

### Forced swimming test (FST)

When the offspring rats reached PND 30, they were divided into four groups according to their sex and maternal group: female offspring in control group (n = 24), male offspring in control group (n = 24), female offspring in CUS group (n = 30), and male offspring in CUS group (n = 30). They were then subjected to the FST. Offspring rats were individually placed in a cylindrical tank measuring 60 cm × 38 cm. The tank was filled with water (24 ± 1°C) at a height of 40 cm. The animals were forced to swim for 15 min (pre-test) and 24 h later they were subjected to a 5 min swimming (test) [24]. Following FST, the rats were removed from the tank, carefully dried in heated cages and then returned to their home cages until decapitation for brain tissue analysis. The total duration of floating and active behaviour (swimming or climbing) was measured during the test. Rats were considered as floating (immobility) when they did not struggle (i.e. they only made movements necessary to keep their heads above the water). Active behavior included vigorous movements of the whole body, active swimming in circles and climbing on the walls of the tank. Increased passive behavioral responses in FST such as immobility and decreased active behaviours, like swimming or climbing, are thought to be a clear indication of “depressive-like” symptomatology [23].

### Blood and brain tissue samples of offspring rats

Offspring rats were anaesthetised with pentobarbital. Before sacrifice, blood samples were taken by cutting the tail to assess serum COR and CRH levels. In order to avoid the influence of circadian cycle on the assessment of serum COR and CRH levels, all blood samples were collected between 10:00 AM and 12:00 AM in our experiment. The rats were decapitated 20 min after the FST session, their brains were rapidly removed and mPFCs from both sides of the brain were isolated on ice for high-performance liquid chromatography (HPLC) and western blot analyses. The brain tissue was dissected to pieces, some of which were used for HPLC and the others were used for western blot analysis.

### Measurement of COR and CRH levels

Blood samples were centrifuged at 4000 rpm for 30 min. The serum was collected and frozen at −70°C until use. Serum COR and CRH levels were determined with a standard radioimmunoassay kit (ICN Biomedicals, Costa Mesa, CA, USA). The sensitivity of the COR radioimmunoassay kit was 20 pg/ml. The inter- and intra-assay coefficients of variation were 7.4% and 9.8% respectively in our experiment. The sensitivity of the CRH radioimmunoassay kit was 40 pg/ml. The intra- and inter-assay variabilities were 6.7% and 8.9%, respectively, in our experiment.

### HPLC procedure

HPLC was used to assay DA and DOPAC. The brain tissue was homogenised and deproteinised in 500 μl of 0.2 N perchloric acid (Merck KgaA, Darmstadt, Germany) solution containing 7.9 mM Na_2_S_2_O_5_ and 1.3 mM disodium ethylenediaminetetraacetic acid (Na_2_EDTA) (both from Riedel-de Haën AG, Seelze, Germany). The homogenate was centrifuged at 14,000 rpm for 30 min in 4°C, and the supernatant was stored at - 80°C. All samples were measured within 1 month; previous studies have shown that monoamines remain stable up to 1 month after homogenisation [25]. The HPLC procedure was performed according to the method previously described [5]. For monoamine analysis, an Agilent HC-C18 analytical column (250 mm × 4.6 mm, 5 μm; Agilent, USA) was used. The mobile phase consisted of 20% methanol and 80% aqueous solution, which contained 30 mM citric acid, 40 mM sodium acetate, 0.2 mM ethylenediaminetetraacetic acid (EDTA) disodium salt and 0.5 mM octanesulphonic acid sodium salt, at a flow rate of 1.0 ml/min and pH 3.8. The level of DA and DOPAC were detected using a Waters 474 scanning fluorescence detector (Waters, USA) with the excitation and emission wavelengths set at 280 nm and 330 nm, respectively. The HPLC system was connected to a computer to quantify all compounds by comparing the area under the peaks with the area of reference standards with specific HPLC software (Chromatography Station for Windows). The turnover ratio of DOPAC to DA is considered as an index of the cell activity causing release of DA, re-uptake, and metabolism to DOPAC [26].

### Western blot analysis

Brain tissues were homogenised in the cell lysis buffer (Beyotime, Jiangsu, China), protein content was determined using the Bio-Rad DC Protein Assay kit (Hercules, CA), and the concentration of each sample was adjusted to 0.3 mg/ml protein. 3 μg of each sample were loaded per lane into 10%-15% SDS-PAGE gels (Invitrogen) for separation by gel electrophoresis. Proteins were subsequently transferred to nitrocellulose (NC) membranes (PerkinElmer Life Sciences, Boston, MA). Blots were incubated in blocking buffer, which was a mixture of 10% non-fat dry milk powder in Tris buffered saline containing 0.5% Tween-20 (TBS-T) for 1 h at room temperature and washed three times for 10 min each in TBS-T. Blots were then incubated at 4°C with the primary antibodies rabbit anti-β-actin (1:2000 dilution; Cell Signalling, USA), rabbit anti-DAT (1:2000 dilution; Cell Signalling, USA), rabbit anti-NET (1:1500 dilution; Cell Signalling, USA), rabbit anti-COMT (1:1000 dilution; Cell Signalling, USA), and washed three times for 10 min each in TBS-T. Blots were cut into 2 parts, high (>50 kDa) and low (<50 kDa) molecular-weight sections, and each was probed with antibodies that recognised proteins of interest within that molecular weight range. Apparent molecular weights for the antibodies used are: β-actin 43 kDa, DAT 68 kDa; NET 68 kDa; COMT 40 kDa. After probing with the primary antibodies, blots were incubated with the appropriate horseradish peroxidase-labelled secondary IgG antibody for 2 h at room temperature, washed three times for 10 min each in TBS-T, and developed with BeyoECL reagents (Beyotime, Jiangsu, China). Band intensity (the sum of the pixels within the band of interest minus the sum of the background pixels) was quantified using Bandscan 5.0 (Glyko Bandscan software). The relative protein levels of DAT, NET and COMT were normalised to β-actin.

### Statistical analysis

Data are presented as mean ± S.E. The data of body weight and sucrose consumption of maternal rats were analysed using one-way repeated measure analysis of variance (ANOVA), with one factor: exposure to CUS (control versus CUS) and one within-subject factor of time (week 0–3). The difference for the moving behavior (total arena, peripheral arena and central arena) in open field test between maternal rats in the CUS group and control group before CUS was analysed using one way ANOVA, and the data of open field test after CUS was performed using covariance analysis. The open field test before and after CUS in the same group was analysed using a paired samples T-test. Statistical analysis of the CRH and COR levels between maternal rats in the CUS and control groups after CUS were analysed using one way ANOVA, and the analyses of CRH and COR levels after delivery were performed using covariance analysis. A two-way ANOVA with two between-subject factors, sex (male versus female) and exposure to CUS (Control versus CUS), was performed for the FST and levels of CRH and COR in the offspring rats. A 3 × 2 ANOVA with three between-subjects factors: sex (male versus female) and exposure to CUS (control versus CUS), and position (left versus right), was used to analyze DA, DOPAC, the ratio of DOPAC to DA, DAT, NET, COMT in the mPFC of the offspring rats. Separate one-way ANOVAs were performed, when needed, in order to elucidate statistical significance between groups. The correlations among behavior in the FST, the CRH or COR levels, the ratio of DOPAC to DA, and levels of DAT, NET, COMT in the mPFC of the offspring rats were analysed by Bivariate correlations. Statistical significance was set at *P* < 0.05.

## Results

### Effect of CUS on body weight and results of sucrose intake test in the maternal rats

**Figure 1:**
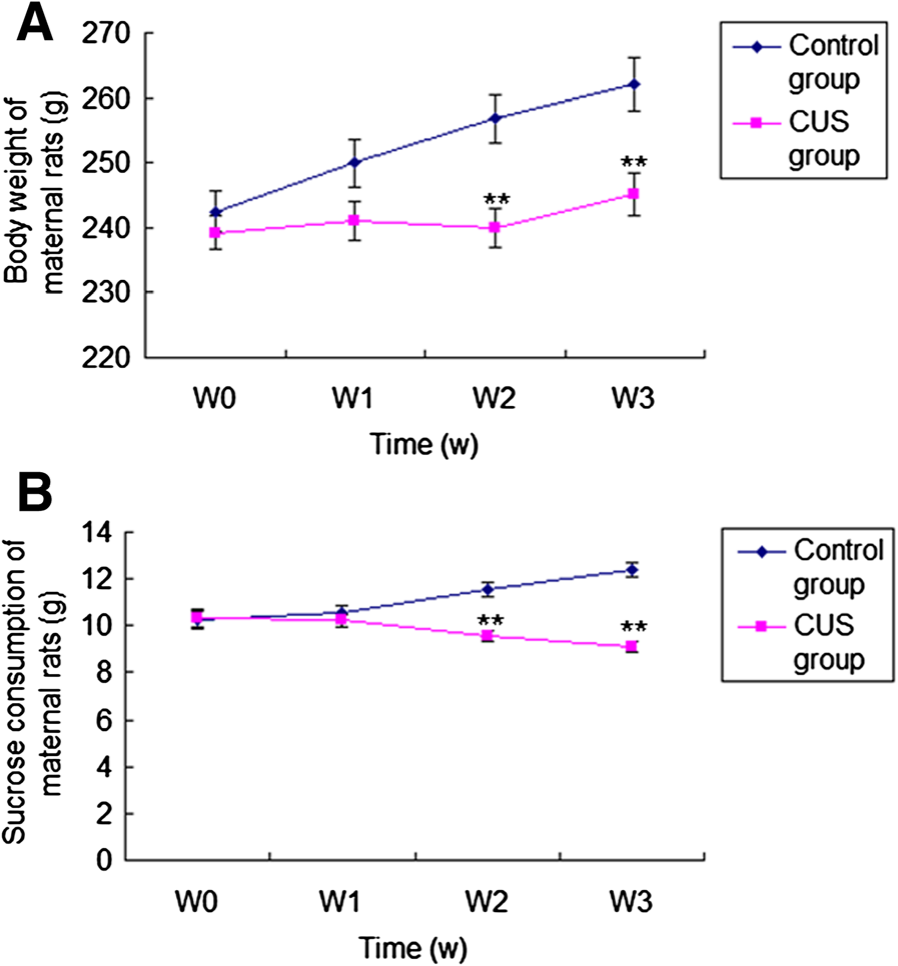
**Effect of CUS on body weight and sucrose consumption in maternal rats.** Body weight **(A)** and sucrose consumption **(B)** in maternal rats from control group (n = 8) and CUS group (n = 12) for weeks (W) 0–3 are shown. Data are the mean ± S.E. **: indicates *P* < 0.01, compared with the control group for the same week.

Figure 1A and 1B show that body weight and sucrose consumption of maternal rats in the CUS group were lower than in the control group at week 2 [body weight: F(1,18) = 11.768, *P* = 0.003; sucrose consumption: F(1,18) =23.720, *P* < 0.001] and week 3 [body weight: F(1,18) = 10.456.385, *P* = 0.005; sucrose consumption: F(1,18) = 77.905, *P* < 0.001]. One-way repeated ANOVA with CUS as the independent factor and time as the repeated factor revealed the effect of CUS on body weight [F(1,18) = 6.385, *P* = 0.021] and sucrose consumption [F(1,18) = 11.334, *P* = 0.003] in maternal rats. The ratio of sucrose consumption per body weight was calculated to examine if the CUS effect on sucrose consumption was related to the changes in body weight. One-way repeated ANOVA with CUS as the independent factor and time as the repeated factor revealed the effect of CUS on the ratio of sucrose consumption per body weight in the maternal rats [F(1,18) = 5.127, *P* = 0.036].

### Effect of CUS on the open field test in the maternal rats

**Figure 2:**
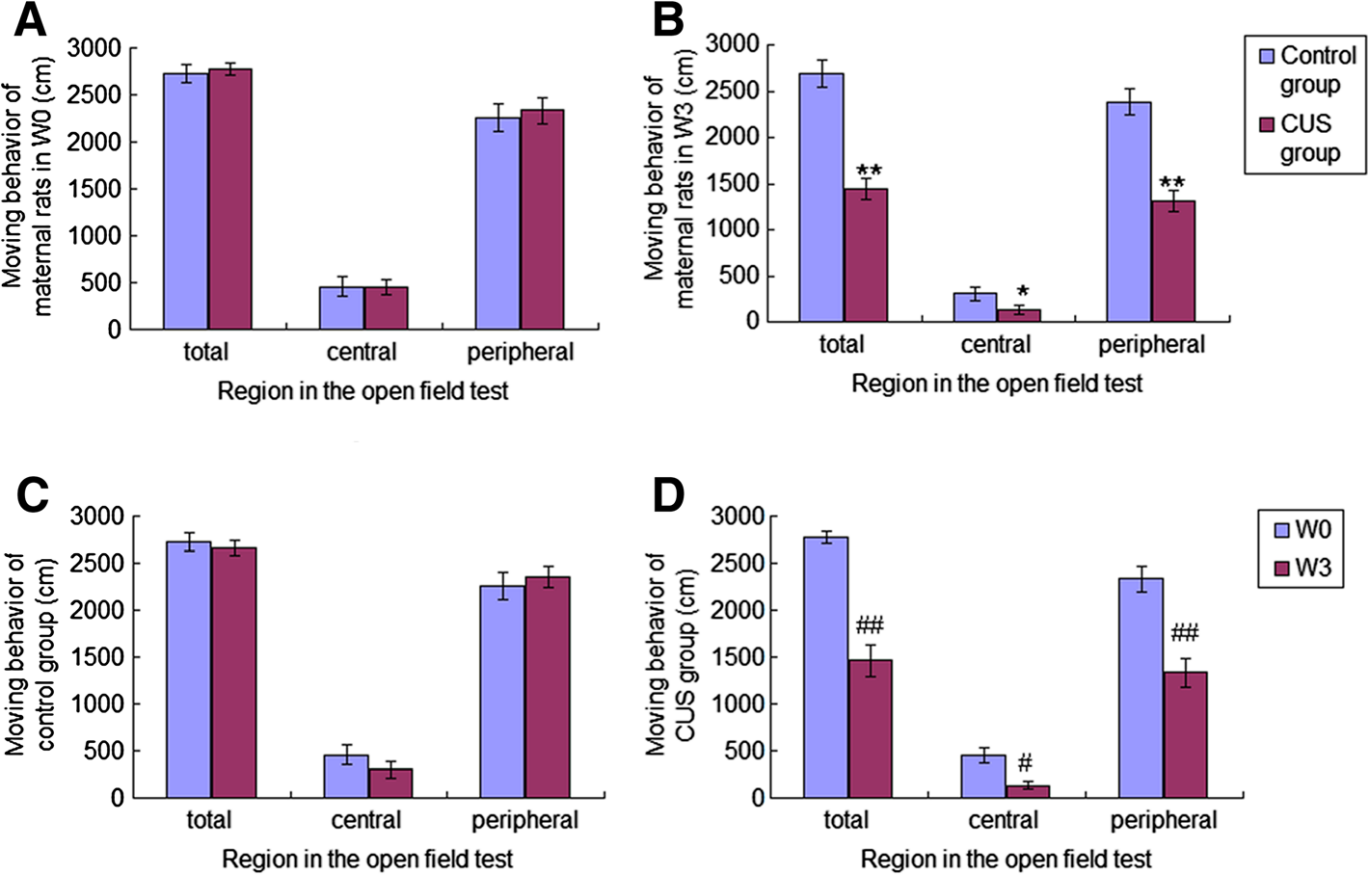
**Moving behaviors of maternal rats in the open field test.** Moving behaviors of maternal rats in the open field test at week 0 (W0) **(A)** and week 3 (W3) **(B)** are shown in Figure 2A and 2B. Moving behaviours of maternal rats from control group **(C**: n = 8**)** and CUS group **(D**: n = 12**)** in the open field test are shown in Figure 2C and 2D. Data are the mean ± S.E. * and ** indicate *P* < 0.05 and *P* < 0.01, respectively, as compared to the control group for the same week. ^#^ and ^##^ indicate *P* < 0.05 and *P* < 0.01, respectively, as compared to W0 for the same group.

There was no difference in the moving behaviour (total arena: F(1,18) = 0.175, *P* = 0.68; peripheral arena: F(1,18) = 0.112, *P* = 0.742; central arena: F(1,18) = 0.04, *P* = 0.949) in the open field test between maternal rats in the CUS group and control group before the CUS procedure (week 0) (Figure 2A). Covariance analysis on the OFT after CUS as the dependent variable and group as the fixed factor and the results of OFT before CUS as covariate revealed that the moving behaviour of maternal rats in the CUS group was decreased significantly compared to the control group at week 3 [total arena: F(1,18) = 42.983, *P* < 0.001; peripheral arena: F(1,18) = 36.616, *P* < 0.001; central arena: F(1,18) = 4.921, *P* = 0.046] (Figure 2B). The reduction of moving behaviour in control maternal rats over 3 weeks did not reach significance (total arena: t = 1.780, df = 7, *P* = 0.118; peripheral arena: t = −1.186, df = 7, *P* = 0.274; central arena: t = 1.968, df = 7, *P* = 0.09) (Figure 2C). However, moving behaviour of maternal rats in the CUS group was decreased at week 3 compared with week 0 (total arena: t = 8.839, df = 11, *P* < 0.001; peripheral arena: t = 6.954, df = 11, *P* < 0.001; central arena: t = 3.070, df = 11, *P* = 0.011) (Figure 2D).

### Effects of pre-gestational CUS on the behavior of offspring rats in the FST

**Figure 3:**
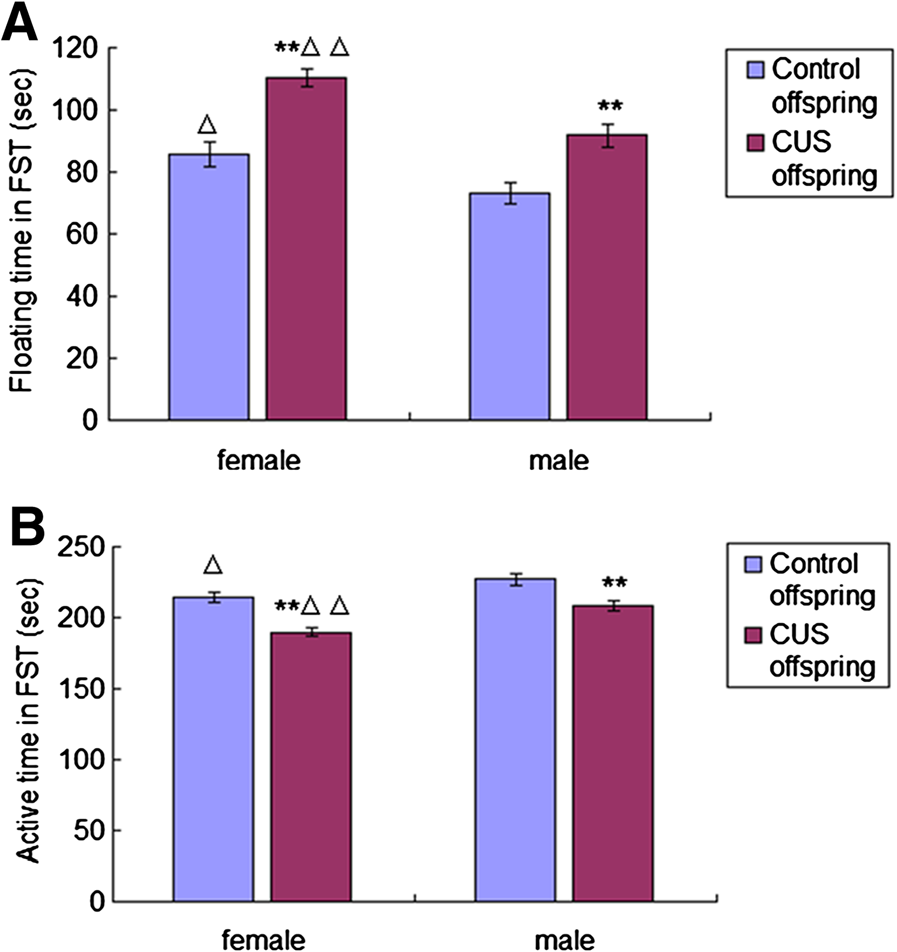
**Effects of pre-gestational stress on performance of offspring rats in the force swimming test.** The floating time **(A)** and active (swimming or climbing) time **(B)** of offspring rats from different groups (female offspring in the control group, n = 24; male offspring in the control group, n = 24; female offspring in the CUS group, n = 30; male offspring in the CUS group, n = 30) in the force swimming test are shown. Data are the mean ± S.E. ** indicates *P* < 0.01 as compared to offspring rats in the control group of the same sex; ^△^ and ^△△^ indicate *P* < 0.05 and *P* < 0.01, respectively, as compared to male offspring rats with the same treatment.

Figure 3A shows that the floating time in FST was longer for offspring rats in the CUS group than controls for both females [F(1,52) = 28.527, *P* < 0.001] and males [F(1,52) = 12.114, *P* = 0.001]. And the floating time in FST was longer in the female offspring rats than in the males for both control [F(1,46) = 5.405, *P* = 0.025] and CUS groups [F(1,58) = 16.027, *P* < 0.001]. The active time (swimming or climbing) in FST was shorter for offspring rats in the CUS group than controls for both females [F(1,52) = 28.527, *P* < 0.001] and males [F(1,52) = 12.114, *P* = 0.001]. Also, the active time in FST was shorter in the female offspring rats than the counterparts males for both control [F(1,46) = 5.405, *P* = 0.025] and CUS groups [F(1,58) = 16.027, *P* < 0.001] (Figure 3B). Two way ANOVA with active time and floating time in FST as dependent variables and CUS and sex as fixed factors revealed effects of CUS [F(3,104) = 37.5, *P* < 0.001], sex [F(3,104) = 19.295, *P* < 0.001] and interaction between CUS and sex [F(3,104) = 0.726, *P* = 0.396] on the active time and the floating time in FST.

### Serum CRH and COR levels in maternal rats and offspring rats

**Figure 4:**
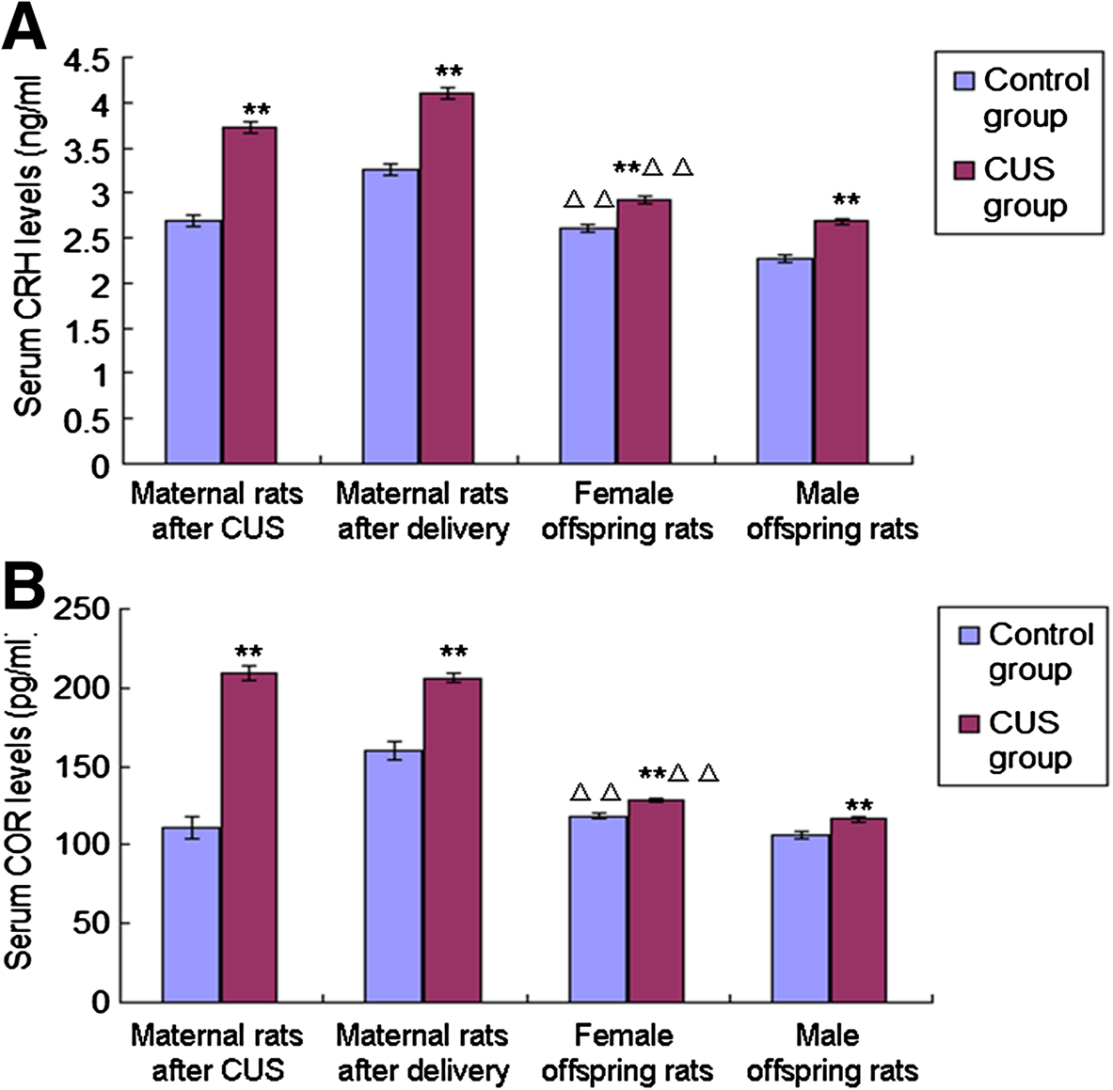
**Effects of pre-gestational stress on CRH and COR levels of maternal rats and offspring rats.** Serum CRH **(A)** and COR **(B)** levels of maternal rats (control group, n = 8; CUS group, n = 12) and offspring rats from different groups (female offspring in the control group, n = 24; male offspring in the control group, n = 24; female offspring in the CUS group, n = 30; male offspring in the CUS group, n = 30) are shown. Data are the mean ± S.E. ** indicates *P* < 0.01 when maternal rats in the CUS group were compared with the control group and offspring rats in the CUS group were compared with the offspring in the control group of the same sex. ^△△^indicates *P* < 0.01, when female offspring rats were compared with male offspring rats of the same treatment.

After the stress procedure, levels of serum CRH [F(1,18) = 106.745, *P* < 0.001] and COR [F(1,18) = 141.711, *P* < 0.001] in the CUS group were significantly higher than in the control group (Figure 4). And after delivery, levels of serum CRH [F(1,18) = 82.506, *P* < 0.001] and COR [F(1,18) = 58.469, *P* < 0.001] in the CUS group were also higher than controls (Figure 4). The covariance analysis using levels of serum CRH and COR after delivery as the dependent variable and group as the fixed factor, and serum CRH and COR after CUS as covariates was performed, which revealed the difference in the levels of serum CRH and COR between CUS group and control group after delivery resulted from the CUS treatment [CRH: F(1,18) = 0.036, *P* = 0.853; COR: F(1,18) = 0.416, *P* = 0.528].

Serum CRH and COR levels were significantly higher in offspring rats in the CUS group than in the control group for both females [CRH: F(1,52) = 23.829, *P* < 0.001; COR: F(1,52) = 21.449, *P* < 0.001] and males [CRH: F(1,52) = 46.979, *P* < 0.001; COR: F(1,52) = 11.758, *P* = 0.001] (Figure 4). Also, levels of serum CRH and COR were higher in the female offspring rats than in the males for both control [CRH: F(1,46) = 23.568, *P* < 0.001; COR: F(1,46) = 17.122, *P* < 0.001] and CUS groups [CRH: F(1,58) = 18.253, *P* < 0.001; COR: F(1,58) = 26.374, *P* < 0.001] (Figure 4). Two way ANOVA using the serum CRH and COR levels as dependent variables and CUS and sex as fixed factors revealed effects of CUS [CRH: F(3,104) = 68.12, *P* < 0.001; COR: F(3,104) = 30.723, *P* < 0.001], sex [CRH: F(3,104) = 42.823, *P* < 0.001; COR: F(3,104) = 42.489, *P* < 0.001], and interaction between CUS and sex [CRH: F(3,104) = 1.321, *P* = 0.253; COR: F(3,104) = 0.012, *P* = 0.912] on the levels of serum CRH and COR.

### DA, DOPAC and the ratio of DOPAC to DA in the left- and right-side mPFCs of offspring rats

3 × 2 ANOVA using levels of DA in the mPFC of offspring rats as a dependent variable and CUS, sex, and position as fixed factors was performed. The results show that levels of DA in the mPFC were not statistically different between CUS and control groups for either female or male offspring rats [F(7,208) = 0.216, *P* = 0.643], and there was no difference in DA levels between the left- and right-side mPFCs for either female or male offspring in the CUS group or control group [F(7,208) = 0.047, *P* = 0.828], nor any difference between female and male offspring rats for either CUS group or control group [F(7,208) = 1.351, *P* = 0.246] (Table 3).

**Table 3:** HPLC analysis of DA, DOPAC, and DOPAC/DA in the left- and right-side mPFCs of the offspring rats

**Groups**	**Sex of offspring**	**Position of mPFC**	**N**	**DA (ng/g)**	**DOPAC (ng/g)**	**DOPAC/DA (%)**
Control	Female	Left	24	202.3 ± 3.11	106.9 ± 1.62	52.8 ± 0.34
		Right	24	199.9 ± 3.7	107.2 ± 1.95	53.7 ± 0.27
Control	Male	Left	24	204.4 ± 3.26	107.5 ± 1.77	52.6 ± 0.34
		Right	24	204.8 ± 3.01	107.9 ± 1.85	52.7 ± 0.43
CUS	Female	Left	30	198.9 ± 2.77	93.9 ± 1.38*	47.3 ± 0.24*
		Right	30	201.3 ± 2.89	76.6 ± 1.58*^#^	36.9 ± 0.49*^#△^
CUS	Male	Left	30	202.7 ± 2.43	97.6 ± 1.34*	48.1 ± 0.29*
		Right	30	202.3 ± 3.06	80.8 ± 1.69*^#^	39.9 ± 0.56*^#^

Data are mean ± S.E. *:*P* < 0.01, compared with controls of the same sex and the same position. ^#^:*P* < 0.01, compared with left-side mPEC for the same group and same sex.^△^:*P* < 0.01, compared with male offspring rats from the same group and same position.

Levels of DOPAC and the ratio of DOPAC to DA in both left- and right-side mPFCs were significantly lower in the offspring rats in the CUS group than in the control group for both females [DOPAC in the left mPFC: F(1,52) = 36.931, *P* < 0.001; DOPAC levels in the right mPFC: F(1,52) = 104.884, *P* < 0.001; DOPAC/DA in the left mPFC: F(1,52) = 187.655, *P* < 0.001; DOPAC/DA in the right mPFC: F(1,52) = 421.137, *P* < 0.001] and males [DOPAC in the left mPFC: F(1,52) = 20.699, *P* < 0.001; DOPAC levels in the right mPFC: F(1,52) = 167.762, *P* < 0.001; DOPAC/DA in the left mPFC: F(1,52) = 100.707, *P* < 0.001; DOPAC/DA in the right mPFC: F(1,52) = 554.276, *P* < 0.001]. The levels of DOPAC and the ratio of DOPAC to DA were lower in the right-side mPFC than in the left-side mPFC of offspring rats in the CUS group for both females [DOPAC: F(1,58) = 36.257, *P* < 0.001; DOPAC/DA: F(1,58) = 145.211, *P* < 0.001] and males [DOPAC: F(1,58) = 103.479, *P* < 0.001; DOPAC/DA: F(1,58) = 387.62, *P* < 0.001], while there was no difference between the two sides of mPFCs of offspring rats in the control group for either females [DOPAC: F(1,46) = 0.019, *P* = 0.89; DOPAC/DA: F(1,46) = 3.519, *P* = 0.067] or males [DOPAC: F(1,46) = 0.016, *P* = 0.899; DOPAC/DA: F(1,46) = 0.003, *P* = 0.957]. The levels of DOPAC were not different between female and male offspring rats in either control group or CUS group for either left-side [control: F(1,46) = 0.082, *P* = 0.776; CUS: F(1,58) = 3.57, *P* = 0.064] or right-side mPFC [control: F(1,46) = 0.06, *P* = 0.807; CUS: F(1,58) = 3.387, *P* = 0.071]. There was no difference in the ratio of DOPAC to DA between female and male offspring rats in the control group for either left-side [F(1,46) = 0.18, *P* = 0.776] or right-side mPFC [F(1,46) = 3.79, *P* = 0.058]. The ratio of DOPAC to DA in the right-side mPFC was lower in the female offspring rats than that of male offspring rats in the CUS group [F(1,58) = 16.51, *P* < 0.001], but there was no difference between them in the left-side mPFC [F(1,58) = 3.48, *P* =0.067] (Table 3).

3 × 2 ANOVA using levels of DOPAC and the ratio of DOPAC to DA as dependent variable and CUS, sex, and position as fixed factors revealed effects of CUS [DOPAC: F(7,208) = 298.224, *P* < 0.001; DOPAC/DA: F(7,208) = 1240.109, *P* < 0.001], sex [DOPAC: F(7,208) = 0.024, *P* = 0.877; DOPAC/DA: F(7,208) = 8.715, *P* = 0.004], position [DOPAC: F(7,208) = 51.799, *P* < 0.001; DOPAC/DA: F(7,208) = 248.493, *P* < 0.001], and interaction among CUS, sex, and position [DOPAC: F(7,208) = 2.852, *P* = 0.093; DOPAC/DA: F(7,208) = 7.695, *P* = 0.006] on DOPAC levels and the ratio of DOPAC to DA in the mPFC of offspring rats.

### Levels of DAT in the left- and right-side mPFCs of the offspring rats

**Figure 5:**
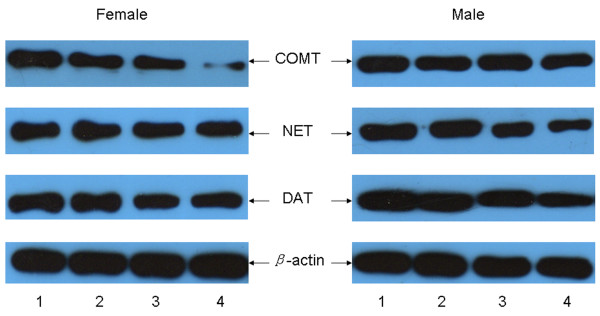
**Western blot analysis levels of DAT, NET, and COMT in the mPFC of both female and male offspring rats.** Lane 1: left-side mPFC of control group females and males, respectively; Lane 2: right-side mPFC of control group females and males, respectively; Lane 3: left-side mPFC of CUS group females and males, respectively; Lane 4: right-side mPFC of CUS group females and males, respectively.

Protein levels of *β*-actin and DAT were detected in the mPEC of offspring rats (Figure 5). The levels of DAT were shown to be lower in the mPFC of offspring rats in the CUS group than in the control group for both females [left: F(1,52) = 44.259, *P* < 0.001; right: F(1,52) = 30.267, *P* < 0.001] and males [left: F(1,52) = 20.262, *P* < 0.001; right: F(1,52) = 26.759, *P* < 0.001]. There was no difference for the relative levels of DAT between the two sides of mPFCs of offspring rats in the control group or CUS group for either females [control: F(1,46) = 0.547, *P* = 0.463; CUS: F(1,58) = 1.099, *P* = 0.299] or males [control: F(1,46) = 0.329, *P* = 0.569; CUS: F(1,58) = 0.057, *P* = 0.785]. The levels of DAT were not different between female and male offspring rats in either left-side or right-side mPFC for either the control group [left: F(1,46) = 0.099, *P* = 0.754; right: F(1,46) = 0.403, *P* = 0.529] or CUS group [left: F(1,58) = 2.638, *P* = 0.11; right: F(1,58) = 0.32, *P* = 0.574] (Table 4).

**Table 4:** Western blot analysis of DAT, NET, and COMT in the left- and right-side mPFCs of the offspring rats

**Groups**	**Sex of offspring**	**Position of mPFC**	**N**	**DAT**	**NET**	**COMT**
Control	Female	Left	24	0.465 ± 0.0067	0.451 ± 0.0055	0.375 ± 0.0057
		Right	24	0.458 ± 0.006	0.461 ± 0.0059	0.368 ± 0.0053
Control	Male	Left	24	0.468 ± 0.0059	0.459 ± 0.0065	0.394 ± 0.0058
		Right	24	0.463 ± 0.0051	0.452 ± 0.0055	0.385 ± 0.0065
CUS	Female	Left	30	0.405 ± 0.0059*	0.351 ± 0.0051*^△^	0.335 ± 0.0067*^△^
		Right	30	0.414 ± 0.0054*	0.320 ± 0.0045*^#△^	0.205 ± 0.0039*^#△^
CUS	Male	Left	30	0.421 ± 0.0079*	0.410 ± 0.0046*	0.381 ± 0.0045
		Right	30	0.418 ± 0.0066*	0.368 ± 0.006*^#^	0.315 ± 0.0038*^#^

Data are mean ± S.E. *:*P* < 0.01, compared with controls of the same sex and the same position. ^#^:*P* < 0.01, compared with left-side mPEC of the same group and same sex.^△^:*P* < 0.01, compared with male offspring rats of the same group and same position.

3 × 2 ANOVA using levels of DAT as a dependent variable and CUS, sex, and position as fixed factors revealed effects of CUS [F(7,208) = 116.658, *P* < 0.001], sex [F(7,208) = 2.516, *P* = 0.114], position [F(7,208) = 0.095, *P* = 0.758], and interaction among CUS, sex, and position [F(7,208) = 0.548, *P* = 0.46] on DAT levels in the mPFC of offspring rats.

### Levels of NET in the left- and right-side mPFCs of the offspring rats

Protein levels of β-actin and NET were detected in the mPEC of offspring rats (Figure 5). Pre-gestational CUS decreased NET levels in both left-side and right-side mPFCs of offspring rats in the CUS group than those in the control group for both females [left: F(1,52) = 32.191, *P* < 0.001; right: F(1,52) = 119.353, *P* < 0.001] and males [left: F(1,52) = 178.348, *P* < 0.001; right: F(1,52) = 351.413, *P* < 0.001]. NET levels were lower in the right-side mPFC than in the left-side mPFC of offspring in the CUS group for both females [F(1,58) = 31.601, *P* < 0.001] and males [F(1,58) = 19.46, *P* < 0.001], while there was no difference between the two sides of mPFCs of offspring in the control group for either females [F(1,46) =1.692, *P* = 0.2] or males [F(1,46) = 0.662, *P* = 0.42]. There was no difference for the NET levels between female and male offspring rats in the control group for either left-side [F(1,46) = 1.058, *P* = 0.309] or right-side mPFC [F(1,46) = 1.154, *P* = 0.288]. The NET levels in both left-side and right-side mPFCs were lower in the female offspring rats than male offspring rats in the CUS group [left: F(1,58) = 75.379, *P* < 0.001; right: F(1,58) = 40.072, *P* < 0.001] (Table 4).

3 × 2 ANOVA using levels of NET as the dependent variable and CUS, sex, and position as fixed factors revealed effects of CUS [F(7,208) = 586.032, *P* < 0.001], sex [F(7,208) = 48.153, *P* < 0.001], position [F(7,208) = 19.959, *P* < 0.001], and interaction among CUS, sex, and position [F(7,208) = 3.65, *P* = 0.057] on NET levels in the mPFC of offspring rats.

### Levels of COMT in the left- and right-side mPFCs of the offspring rats

Protein levels of β-actin and COMT were detected in the mPEC of offspring rats (Figure 5). The COMT levels in the right mPFC of offspring rats in the CUS group were lower than those in the control group for both females [F(1,52) = 301.231, *P* < 0.001] and males [F(1,52) = 481.606, *P* < 0.001]. Levels of COMT in the left mPFC of female offspring rats in the CUS group were lower than those in the control group [F(1,52) = 41.872, *P* < 0.001], but there was no difference in the left mPFC of male offspring between the CUS group and the control group [F(1,52) = 0.783, *P* = 0.38]. COMT levels were lower in the right-side mPFC than in the left-side mPFC of offspring in the CUS group for both females [F(1,58) = 538.69, *P* < 0.001] and males [F(1,58) = 285.928, *P* < 0.001], but there was no difference between the two sides of mPFCs of offspring in the control group for either females [F(1,46) = 2.183, *P* = 0.107] or males [F(1,46) = 2.362, *P* = 0.139]. There was no difference for the COMT levels between female and male offspring rats in the control group in either left-side [F(1,46) = 3.97, *P* = 0.059] or right-side mPFC [F(1,46) = 1.21, *P* = 0.277]. The COMT levels in both left-side and right-side mPFCs were lower in the female offspring rats than male offspring rats in the CUS group [left: F(1,58) = 32.493, *P* < 0.001; right: F(1,58) = 54.577, *P* < 0.001] (Table 4).

3 × 2 ANOVA using levels of COMT as dependent variable and CUS, sex, and position as fixed factors revealed effects of CUS [F(7,208) = 456.801, *P* < 0.001], sex [F(7,208) = 14.374, *P* < 0.001], position [F(7,208) = 437.596, *P* < 0.001], and interaction among CUS, sex, and position [F(7,208) = 1.119, *P* = 0.291] on COMT levels in the mPFC of offspring rats.

### Correlations

Correlations among serum COR and CRH levels, the ratio of DOPAC to DA, and levels of DAT, NET, COMT in the mPFC were analysed by the Bivariate correlation analysis. Offspring rats with a low ratio of DOPAC to DA in the mPFC exhibited a longer duration of passive behavior (floating) in the FST (*r* = −0.42, *P* < 0.01). Offspring rats with higher CRH and COR levels had a lower ratio of DOPAC to DA (CRH: *r* = −0.533, *P* < 0.01; COR: *r* = −0.423, *P* < 0.01). Decreased DAT expression (CRH: *r* = −0.361, *P* < 0.01; COR: *r* = −0.294, *P* < 0.01) in the mPFC also exhibited a longer duration of passive behavior (floating) in the FST (CRH: *r* = 0.459, *P* < 0.01; COR: *r* = 0.398, *P* < 0.01). Offspring rats with higher CRH had a decreased NET expression (*r* = −0.275, *P* < 0.01) in both left- and right-side mPFCs, but the correlation between COR levels and NET expression (*r* = −0.241, *P* < 0.05) existed only in the right mPFC. Offspring rats with higher CRH and COR levels had a decreased COMT expression (CRH: *r* = −0.486, *P* < 0.01; COR: *r* = −0.318, *P* < 0.01) in the right-side mPFC, but the correlation did not exist in the left mPFC. There were positive correlations between the ratio of DOPAC to DA and levels of DAT, NET and COMT in both left- and right-side mPFCs. The offspring rats of CUS with lower DAT, NET, COMT expression in the mPFC had a lower ratio of DOPAC to DA (DAT: *r* = 0.455, *P* < 0.01; NET: *r* = 0.719, *P* < 0.01; COMT: *r* = 0.809, *P* < 0.01).

## Discussion

The present study was designed to elucidate whether pre-gestational stress could affect offspring on the mPFC dopaminergic activity in response to acute stress in puberty and if so, whether such effects exhibited hemispheric asymmetry or sexual dimorphism. These data indicate that the dopaminergic activity of offspring rats in response to acute stress was affected by the pre-gestational CUS of maternal rats, the sex of the offspring rats, and the position or sideness of the mPFC. Pre-gestational CUS decreased dopaminergic activity as well as levels of DAT, NET and COMT in the mPFC of offspring rats. Such reductions were lateralised to the right hemisphere. Furthermore, the offspring rats of CUS group exhibited sexual dimorphism in the dopaminergic neurotransmission when they expose to acute stress such as FST.

### Pre-gestational CUS-induced behavioral changes in maternal rats

We had established and assessed the CUS model of maternal rats. Gain of body weight in maternal rats exposed to CUS was significantly slower than in controls. In the sucrose intake test, control maternal rats increased their consumption of sucrose solution over the period of three weeks while maternal rats exposed to CUS showed a less sucrose consumption. The open field test showed that the moving behavior of CUS and control maternal rats before CUS was not significantly different. However, the CUS treatment significantly reduced the moving behavior (relative to the control group and their own pre-CUS behavior) after 21 days. The body weight and behavior findings for maternal rats were in agreement with our previous findings [4,5], thus the CUS model was suitable for our study.

### Pre-gestational CUS-induced changes in serum COR and CRH levels in maternal rats and offspring rats

The serum COR and CRH levels were higher in maternal rats with pre-gestational CUS than in the control group both after CUS and delivery. This finding may relate to the dysregulation of the HPA axis induced by CUS, which is supported by previous studies [4,5,27,28]. Chronic stress not only leads to the imbalance of the neuro-endocrine network of maternal rats, but also impacts on the HPA axis of fetus via maternal-placental-foetal interface [29,30]. The early stage of pregnancy is the most sensitive period in which stress affects neuro-behavioral changes in the offspring. Imposing CUS on maternal rats for a short time before pregnancy may also affect the fetal environment and thus produce a profound effect on the brain development of offspring [4,5]. During the embryonic developement, the brain undergoes rapid growth that is characterised by a high turnover rate of neuronal connections. Therefore, the fetal brain is especially vulnerable to excess hormonal influence from the maternal circulation such as under stress conditions [31]. In our study, offspring rats were forced to swim for a 15 minutes, and after a 24-hour interval, they were subjected to a 5-minute FST. Blood samples of offspring rats were taken post FST. We found that serum CORT and CRH levels were significantly higher for offspring rats in the CUS group than those in the control group, which may be related to pre-gestational stress history induced dysfunctional HPA responsivity to the acute stressor (FST) in offspring rats. In a word, not only chronic elevation of COR and CRH but also dysfunctional HPA responsivity to the acute stressor could impact on a balanced supply of stress hormones which appears to be important for development of the nervous system and the behavior in the offspring [32]. It is well known that stress hormones can modulate behaviors and facilitate DAergic neurotransmission in brain regions, especially upon pharmacological challenge or during conditions of neural activation [33-35].

### Pre-gestational CUS-induced alterations in the dopaminergic system in the mPFC of offspring rats

Both female and male offspring rats born to the CUS mothers exhibited reduced behavioral activity during pre-test session and produced a longer duration of passive behavior (floating) in the FST compared to offspring rats in the control group. This finding indicates an impact of pre-gestational stress on FST performance of offspring rats, which could be attributed to a poor coping strategy. Our findings also show that pre-gestational stress induced a decreased DOPAC levels and the ratio of DOPAC to DA in the mPFC of offspring rats in the CUS group than in the control group. The results were significantly correlated with the altered in the FST observed. The differences, however, were more pronounced in the right hemisphere.

Of note, although pre-gestational CUS caused a decreased in the activation of mPFC dopaminergic system that was biased toward the right hemisphere of the offspring rats in the CUS group, such bias was not observed in the offspring rats in control group. This is a novel observation, given that such “symmetric” left–right mPFC dopaminergic activity in responses to acute stress were seen in the offspring rats of control group. Thus, taken together with our previous findings, the present data indicate that exposure to pre-gestational CUS leads to an asymmetric (right-sided) attenuation of mPFC dopaminergic activity in response to acute stress in the offspring rats during puberty (PND 30).

Impaired mPFC function, particularly when it is lateralised to the right hemisphere, has been linked to a number of stress-related pathologies [20,22]. In animals, right-sided mPFC dopaminergic activity is associated with responding to novel stressful environments [36], protection from stress ulcer pathology [37], and escape after exposure to uncontrollable shock [38]. In humans, studies reveal that left-biased frontal electroencephalographic (EEG) asymmetry is associated with approach behaviours and positive effects, whereas right-sided biases are linked to withdrawal and defensive behavior. Neuroimaging studies show that the right ventromedial mPFC is closely correlated with negative effects [39]. Moreover, alterations in autonomic function and cognitive–emotional processing resulting from insult to the ventromedial mPFC appear to be attributable primarily to the right hemisphere damage [40].

It is likely that an altered dopaminergic activity resulted in part from the differences in the expression of DAT in the mPFC. DAT is present throughout fetal development and expressed at high levels during neurogenesis and rapid brain growth [41]. DA levels within the synaptic cleft are regulated by the activity of the DAT which re-uptakes DA into the pre-synaptic terminals and therefore regulates the duration of the DA activity [42]. DAT is a key regulator of dopamine homeostasis in synapses and plays an important role in regulating behaviors governed by the dopaminergic system [43,44]. With higher levels of DAT, extracellular DA could presumably be taken-up more rapidly for subsequent release [45,46]. Therefore, the finding that the levels of DAT in the left- and right-side mPFCs of offspring rats in the CUS group were both lower would be consistent with the bilateral attenuation of the dopaminergic activity seen in these animals. This explanation, however, could not account for the difference in the levels of DAT between the two sides of mPFCs of offspring rats in the CUS group. Given the weaker rightward bias of the mPFC dopaminergic activity in response to acute stress in these animals, one would expect to find hemispheric asymmetries in DAT levels in the mPFC. However, this was not what we observed. In the offspring rats of the CUS group, left and right mPFC DAT levels were equally reduced relative to those in the control group. Clearly, other mechanisms are involved.

One likely mechanism would be the norepinephrine (NE) input to the mPFC, which is known to be activated by stress and appears to regulate DA-mediated function [47]. In the mPFC, for instance, DA re-uptake appears to be mediated as much, if not more, by the NET than by the DAT [48]. Thus, it is likely that some of the group and hemispheric differences in the mPFC dopaminergic activity in response to acute stress might reflect alterations in NET levels. Our findings in this study support this hypothesis. Another potentially important mechanism involves COMT. COMT is a postsynaptic enzyme that methylates DA and, in the mPFC, appears to be the primary mechanism by which extracellular DA is inactivated [49]. Studies in humans suggest that a functional polymorphism in the COMT gene is associated with altered performance in mPFC-mediated cognitive tasks [50]. Variants in the COMT gene may be associated with susceptibility to schizophrenia [51], which involves an altered dopaminergic activity in the mPFC. With higher levels of COMT, extracellular DA would presumably be degraded more rapidly, resulting in more DA metabolised to DOPAC [26]. The extracellular DOPAC concentration is altered in response to neuronal activity, which essentially reflects an increased dopaminergic neuron activity [52]. In this study, we found that levels of COMT in the left mPFC of female offspring rats in the CUS group were lower than those in the control group, but there was no difference in the male offspring between the CUS group and control group. Also, the difference in attenuated COMT levels between the two sides of mPFCs of offspring rats in the CUS group was significant for both females and males. These findings would implicate the role of COMT in the group and hemispheric differences in the mPFC dopaminergic activity observed here.

### Sex differences in the effect of pre-gestational CUS on the dopaminergic activity response to acute stress in the offspring rats

It has been demonstrated that females respond differently during the application of stressful regimens, a fact that underscores the critical role of gender differences in stress-related disorders [53-56]. Dalla et al. [33] found that female rats had higher immobility duration than males and that the dopaminergic activity was decreased in the PFC of female rats following chronic mild stress but enhanced in males following FST, which generally agrees with our findings. In the present study, sex differences in the effect of pre-gestational stress on the mPFC dopaminergic activity in response to acute stress (i.e. FST), as reflected by the ratio of DOPAC to DA and the levels of NET and COMT, were only observed in the offspring rats from CUS group, indicating that this phenomenon was attributed to the pre-gestational CUS in the maternal rats.

The “depressive-like” profile induced by acute stress (i.e. FST) was more pronounced in female offspring rats than males in both the control and CUS groups, since floating time was longer and swimming/climbing time was shorter in female offspring rats as compared to males. The longer swimming/climbing time in male offspring might be related to the enhanced dopaminergic activity in response to FST, since swimming/climbing has been considered a behavioral measure of noradrenergic [57] and dopaminergic neurotransmission [58]. The activation of the mPFC dopaminergic system has been considered as an aspect of optimal cognitive function [59]. Additionally, the lack of mPFC dopaminergic activation in female offspring in response to FST may reflect their inability to cope with the stressful procedure. This lack could be due to the fact that female offspring rats in both control and CUS group had higher stress hormone levels than their male counterparts. It is possible that the dopaminergic system cannot be further activated in the female offspring from the CUS group when they were subjected to acute stress (i.e. FST).

The most striking effects of pre-gestational CUS observed in the present study were the gender difference in tissue NET and COMT contents, as well as DA turnover ratios (male biased). These observations are consistent with behavioral changes in the offspring rats from the CUS group. Given that DA conversion to DOPAC is primarily intracellular following reuptake, the difference in reuptake kinetics may partially explain the sex differences. Some studies have reported that stress hormones reduce DA reuptake or DAT sensitivity [60]. However, the results of our study have shown that the levels of DAT were not different between female and male offspring rats in either left-side or right-side mPFC for either the control group or the CUS group. Therefore, DAT sensitivity is unlikely to explain the present results. Additional mechanisms may account for these sex differences. In the terminal regions where DA function is less tightly self-regulated by DAT and autoreceptors than in striatum [61]. In mPFC, DA uptake appears to be mediated as much by the NET. As such, male offspring rats from CUS had relatively more reuptake in dopaminergic terminals, hence higher DA turnover ratios in males than females observed in our study. Sex effects have also been reported in COMT levels of mPFC. Consistent with our findings in behavioral changes and DA turnover ratios, female offspring rats in CUS group have been shown to have lower tissue concentrations of COMT in mPFC than males.

## Conclusion

In the offspring rats born to pre-gestational CUS-treated mothers, females exhibited a lower DA metabolism than males in the mPFC, a brain region known to regulate stress and emotion processing. Also, pre-gestational CUS resulted in both sex-specific and position-specific (left- or right-side of mPFC) deregulation of the dopaminergic activity in response to acute stress in the pubertal offspring rats. It thus implies that sex differences contribute to the pre-gestational CUS on the dopaminergic activity. The observation may underlie differential gender vulnerability to stress and emotional disorders seen clinically. However, the functional implications of the lateralisations to the dopaminergic activity in response to acute stress in pubertal offspring remain to be defined. Moreover, the precise mechanism by which pre-gestational CUS regulates the development of prefrontal dopaminergic systems is yet to be clarified. We will address these questions in our forthcoming studies.

## Abbreviations

COMT: Catechol-omethyltransferase; COR: Corticosterone; CUS: Chronic unpredictable stress; CRH: Corticotrophin releasing hormone; DA: Dopamine; DAT: Dopamine transporter; DOPAC: Dihydroxy-phenyl acetic acid; EDTA: Ethylenediamine tetraacetic acid; FST: Forced swimming test; GABA: γ-aminobutyric acid; GCs: Glucocorticoids; Glu: Glutamate; HPA: Hypothalamic-pituitary-adrenal; HPLC: High-performance liquid chromatography; mPFC: medial prefrontal cortex; NE: Norepinephrine; NET: Norepinephrine transporter; PND: Postnatal day.

## Competing interests

The authors declare that they have no competing interests in our study.

## Authors’ contributions

HYJ and CSH are co-first authors, and they made equal contributions to this work. HYJ performed the Western blot analysis and drafted the manuscript. CSH performed the radioimmunoassay analysis and helped to carry out the statistical analysis. XHW carried out the HPLC procedure and helped to draft the manuscript. LHH participated in designing the experimental animal model. YXC performed the statistical analysis. HGY helped to draft the manuscript. SXC and HQJ are co-corresponding authors, and they made equal contributions to this work. SXC conceived of the study and participated in its design. HQJ participated in the overall design of the study. All authors read and approved the final manuscript.
